# Astrocytes Excessively Engulf Synapses in a Mouse Model of Alzheimer’s Disease

**DOI:** 10.3390/ijms25021160

**Published:** 2024-01-18

**Authors:** Lingjie Li, Shuai Lu, Jie Zhu, Xiaolin Yu, Shengjie Hou, Yaru Huang, Xiaoyun Niu, Xiaoyu Du, Ruitian Liu

**Affiliations:** 1 State Key Laboratory of Biochemical Engineering, Institute of Process Engineering, Chinese Academy of Sciences, Beijing 100190, China; 2 University of Chinese Academy of Sciences, Beijing 100049, China; 3 College of Life Science, Ningxia University, Yinchuan 750021, China

**Keywords:** astrocytes, Alzheimer’s disease, synapse elimination, synapse engulfment, multiple EGF-like domains 10, lysosomal-associated membrane protein 1

## Abstract

Synapse loss is one of the most critical features in Alzheimer’s disease (AD) and correlates with cognitive decline. Astrocytes mediate synapse elimination through multiple EGF-like domains 10 (MEGF10) pathways in the developing and adult brain to build the precise neural connectivity. However, whether and how astrocytes mediate synapse loss in AD remains unknown. We here find that the phagocytic receptor MEGF10 of astrocytes is significantly increased in vivo and in vitro, which results in excessive engulfment of synapses by astrocytes in APP/PS1 mice. We also observe that the astrocytic lysosomal-associated membrane protein 1 (LAMP1) is significantly elevated, colocalized with the engulfed synaptic puncta in APP/PS1 mice, and astrocytic lysosomes contain more engulfed synaptic puncta in APP/PS1 mice relative to wild type mice. Together, our data provide evidence that astrocytes excessively engulf synapses in APP/PS1 mice, which is mediated by increased MEGF10 and activated lysosomes. The approach targeting synapse engulfment pathway in astrocytes would be a potent therapy for AD.

## 1. Introduction

Alzheimer’s disease (AD) is characterized by synapse loss and neuronal degeneration, progressive cognitive decline in older individuals, and the accumulation of aggregates of beta amyloid (Aβ) and hyperphosphorylated tau in the brains [1]. During development, microglia play a crucial role in the refinement of neuronal circuits through the engulfment of excess synapses via a classical complement-dependent pathway [2]. In the progress of AD, Aβ exhibits a preference for binding to synapses, thereby initiating complement generation and triggering microglia to excessively eliminate synapses through the complement-dependent pathway, ultimately leading to synapse loss and cognitive deficits [3,4].

Astrocytes are abundant cells in the central nervous system (CNS), constituting approximately 20% to 40% of the total number of human brain cells. Astrocytes play a prominent role in providing trophic support for neurons, facilitating the development and function of synapses, pruning synapses through phagocytosis, and carrying out various other functions related to maintaining homeostasis [5,6,7,8,9,10]. Previous study demonstrated that astrocytes were enriched in genes for phagocytic pathways, including two phagocytic receptors, namely multiple EGF-like domains 10 (*Megf10*) and macrophage c-mer tyrosine kinase (*Mertk*) [11]. In the developing brain, astrocytes contribute to activity-dependent synapse pruning and developmental refinement of circuits by phagocytosing synapses through phagocytic receptors MEGF10 and MERTK [6]. In the adult hippocampus, astrocytes eliminate unnecessary excitatory synaptic connections through MEGF10 [12]. Moreover, previous studies have shown that inhibition of the astrocyte-engulfment process leads to accumulation of dysfunctional synapses leading to memory impairments in mice [12,13]. Thus, astrocytes are crucial for maintaining circuit connectivity and thereby supporting cognitive function. However, whether synapse elimination induced by astrocytes contributes to the synapse loss in AD remains unknown.

Lysosomes are prominent organelles within cells responsible for degradation, housing approximately 25 membrane proteins and over 60 soluble proteins in their lumen [14]. Among these luminal proteins, the majority are acid hydrolases by which various macromolecules are degraded and recycled, including proteins, lipids, polysaccharides, and nucleic acids. These macromolecules are trafficked to the lysosomes via several pathways, such as autophagy, phagocytosis, and endocytosis [14,15]. Lysosomal-associated membrane protein 1 (LAMP1) is routinely used to label endo-lysosomal vesicles. Multiple studies have shown a higher presence of lysosomes and/or lysosomal proteins surrounding amyloid plaques [16,17]. Lysosomal dysfunction in AD has been wildly studied [18,19]. Since phagocytosis and endo-lysosomes are integrated cellular homeostasis processes, it is our interest to understand whether lysosomes participate in the synapse elimination induced by astrocytes in AD mice. In this study, we detected the synapse engulfment by astrocytes in APP/PS1 mice and investigated the corresponding mechanism.

## 2. Results

### 2.1. Loss of Synapses in APP/PS1 Mice

**Figure 1:**
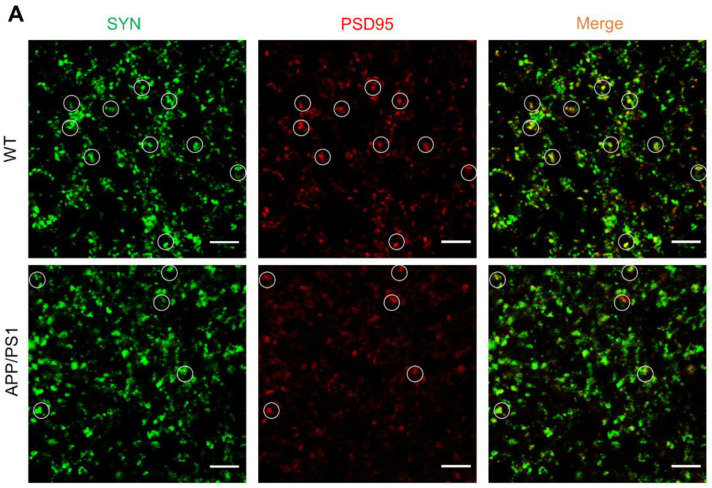

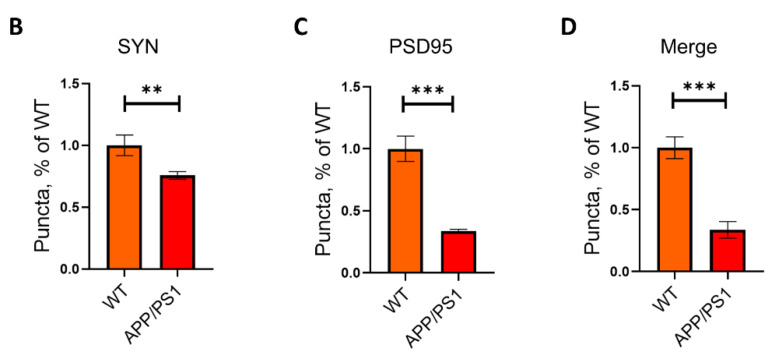
Loss of synapses in APP/PS1 mice. (**A**) PSD95 immunostaining and synaptophysin (SYN) immunostaining in the brains of APP/PS1 mice and their WT littermates. White circles indicate the colocalized PSD95 with SYN puncta. (Scale bar: 5 μm). (**B**–**D**) Quantification of synaptophysin (SYN) puncta (**B**), PSD95 puncta (**C**) and their apposition (**D**) in (**A**). *n* = 5 mice per group. Data represent means ± SEM and an unpaired *t* test with two-tailed was used for statistical analysis. (**B**–**D**) **, *p* < 0.01; ***, *p* < 0.001.

Synapse loss is a key feature of AD pathology, which is strongly linked to cognitive deficit [20]. The six-months old APP/PS1 mice, which have amyloid plaques and cognitive impairment, are useful in studying neurological disorders of the brain, specifically AD. To assess the synapse density of APP/PS1 mice and their WT littermates, we measured the levels of two synaptic marker proteins, synaptophysin (SYN) and postsynaptic density protein-95 (PSD95) in the hippocampus of APP/PS1 mice by immunohistochemistry (Figure 1A). The colocalization of pre- and postsynaptic markers represent structural integrity of synapses, quantification of colocalized pre- and postsynaptic puncta (synaptophysin and PSD95) revealed a significant decrease of synapses in APP/PS1 (Figure 1B–D), suggesting that synapse loss in APP/PS1 mice.

### 2.2. Astrocytes Excessively Engulf Synapses in APP/PS1 Mice

**Figure 2:**
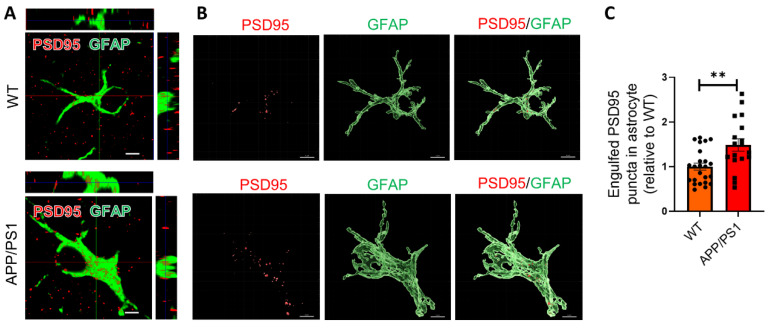
Astrocytes excessively engulf synapses in APP/PS1 mice. (**A**) Orthogonal view of high-resolution confocal image of colocalization of PSD95 (red) and GFAP (green) in the hippocampus of WT mice and APP/PS1 mice. Scale bar: 3 μm. (**B**) Three-dimensional reconstruction of PSD95 (red) puncta inside astrocytes (green) in the hippocampus of APP/PS1 mice and WT mice via Imaris (Version 9.8). Scale bar: 3 μm. (**C**) Quantification of PSD95 puncta in astrocytes in (**B**) by Image J (Version 1.52a). *n* = 3 mice per group, 6 to 8 astrocytes analyzed per mouse. Data are mean ± SEM, and an unpaired *t* test with two-tailed was used for statistical analysis. **, *p* < 0.01.

The decline of cognitive function in AD is more strongly correlated with synapse loss compared to the plaques, tau protein tangles, and neuronal loss [20]. It has been reported that microglia mediate early synapse loss in AD mouse models via a complement pathway, causing cognitive impairment [4,21]. As astrocytes have the physiological ability to prune synapse, we wonder whether astrocytes are also involved in the excessive synapse elimination. To determine the hypothesis, we performed immunohistochemistry (IHC) to evaluate the amount of PSD95, a neuronal synaptic marker protein, in GFAP-immunoreactive astrocytes in the hippocampus of APP/PS1 mice and their wild-type (WT) controls. Our results revealed that astrocytes exhibited a significantly higher volume of internalized PSD95 puncta (about 1.5-fold increase) per cell in the hippocampus of APP/PS1 mice compared with WT mice, indicating that astrocytes ingested more synapses in APP/PS1 mice (Figure 2A–C).

### 2.3. Astrocytes Show High Levels of LAMP1 Immunoreactivity in APP/PS1 Mice

**Figure 3:**
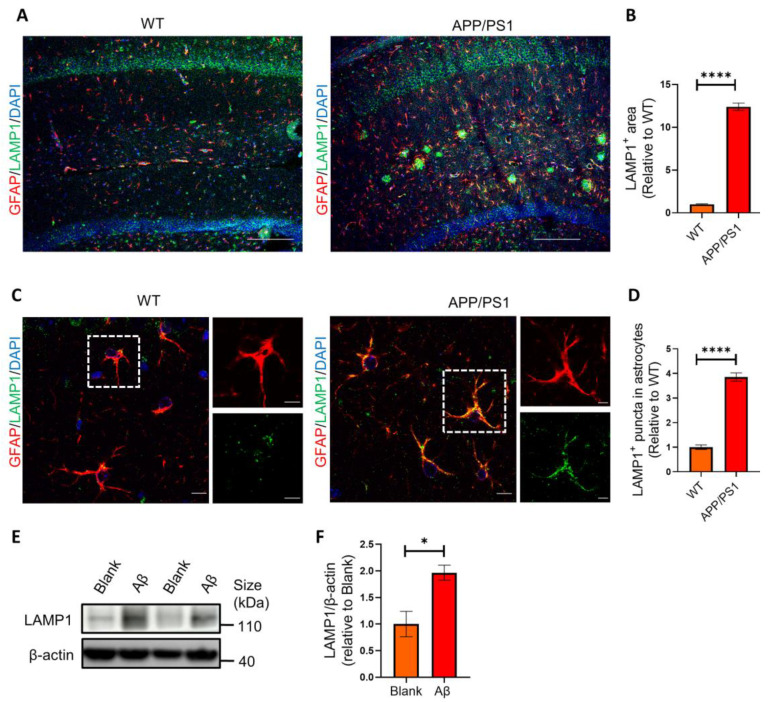
Astrocytes show high levels of LAMP1 immunoreactivity both in vivo and in vitro. (**A**) Representative confocal images of LAMP1^+^ (green) immunoreactivity and GFAP^+^ (red) astrocytes in APP/PS1 mice and WT mice. Scale bar: 250 μm. (**B**) Quantification of LAMP1^+^ area in (**A**) by Image J. *n* = 5 mice per group. (**C**) Representative confocal images of LAMP1^+^ lysosomes (green) in GFAP^+^ (red) astrocytes in APP/PS1 mice and WT mice. Representative astrocytes in white rectangles are further magnified. Scale bar: 10 μm (left) and 5 μm (right). (**D**) Quantification of LAMP1^+^ puncta in astrocytes in (**C**) by Image J. *n* = 5 mice per group. (**E**) Western blots analysis of LAMP1 in primary astrocytes challenged with AβOs. (**F**) Quantification of protein bands in (**E**) by ImageJ. Data are pooled from three independent experiments. Data in B, D and F are mean ± SEM, and an unpaired *t* test with two-tailed was used for statistical analysis. *, *p* < 0.05; **** *p* < 0.0001.

LAMP1 is a major endosomal/lysosomal membrane protein that is thought to be responsible for lysosomal function in phagocytosis. To determine whether the internalized synapses were targeted to lysosomes, we first examined the amount of LAMP1^+^ lysosomes in astrocytes in the hippocampus of mice. We found that the LAMP1^+^ area (Figure 3A,B) and amount of LAMP1^+^ lysosomes in astrocytes were markedly increased in APP/PS1 mice (Figure 3A–D). These results demonstrated that the astrocytic lysosomes were significantly activated in APP/PS1 mice.

To further investigate the effect of Aβ on lysosomes and mimic pathological microenvironment possessing higher Aβ levels in the brains of AD patients, we added Aβ oligomers (AβOs) to primary hippocampal astrocytes culture. Our Western blotting results showed that the protein level of lysosomal markers LAMP1 was significantly upregulated in the cultured astrocytes with AβOs treatment (Figure 3E,F).

### 2.4. Phagocytosed Synapses Are Located in Astrocytic Lysosomes

**Figure 4:**
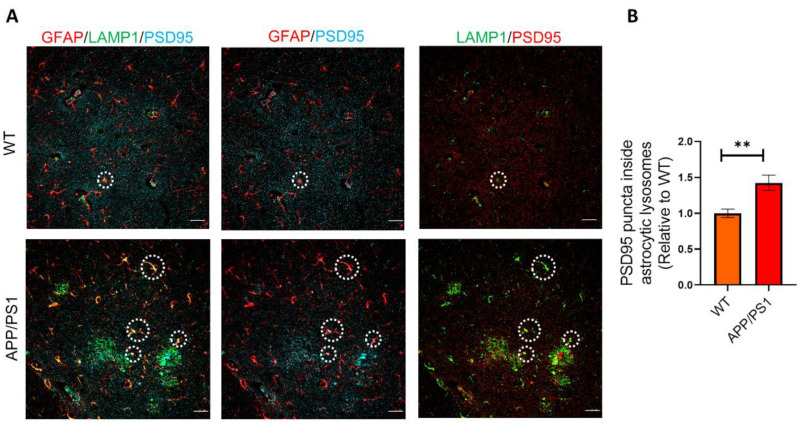
The phagocytosed synapses are located in astrocytic lysosomes. (**A**) Left: Representative confocal images of colocalization of LAMP1^+^ lysosomes (green) and internalized PSD95^+^ puncta (cyan) in GFAP^+^ astrocytes (red) in APP/PS1 mice and WT mice. Middle: Representative confocal images of colocalization of internalized PSD95 puncta (cyan) and GFAP^+^ astrocytes (red) in APP/PS1 mice and WT mice. Right: Representative confocal images of colocalization of internalized PSD95 puncta (red) and LAMP1^+^ lysosomes (green) in astrocytes in APP/PS1 mice and WT mice. Representative astrocytes are indicated by white circles. Scale bar: 25 μm. (**B**) Quantification of engulfed PSD95 puncta in astrocytic lysosomes in APP/PS1 and WT mice by Image J. *n* = 5 mice per group. Data are mean ± SEM, and an unpaired *t* test with two-tailed was used for statistical analysis. ** *p* < 0.01.

To determine the localization of the engulfed PSD95^+^ synapses in astrocytes, we detected synapse marker PSD95 and LAMP1^+^ lysosomes in astrocytes by immunostaining. The results showed that astrocytic LAMP1^+^ lysosomes contained more engulfed PSD95^+^ synapses puncta in APP/PS1 mice relative to WT mice, suggesting that the engulfed synapses were digested via lysosome pathway (Figure 4A,B). Besides, we found the number of PSD95^+^ excitatory post-synapse puncta were substantially reduced in AD model mice (Figure 4A), which was consistent with the feature of AD mice.

### 2.5. The Phagocytic Receptor MEGF10 Is Significantly Increased Both In Vivo and In Vitro

**Figure 5:**
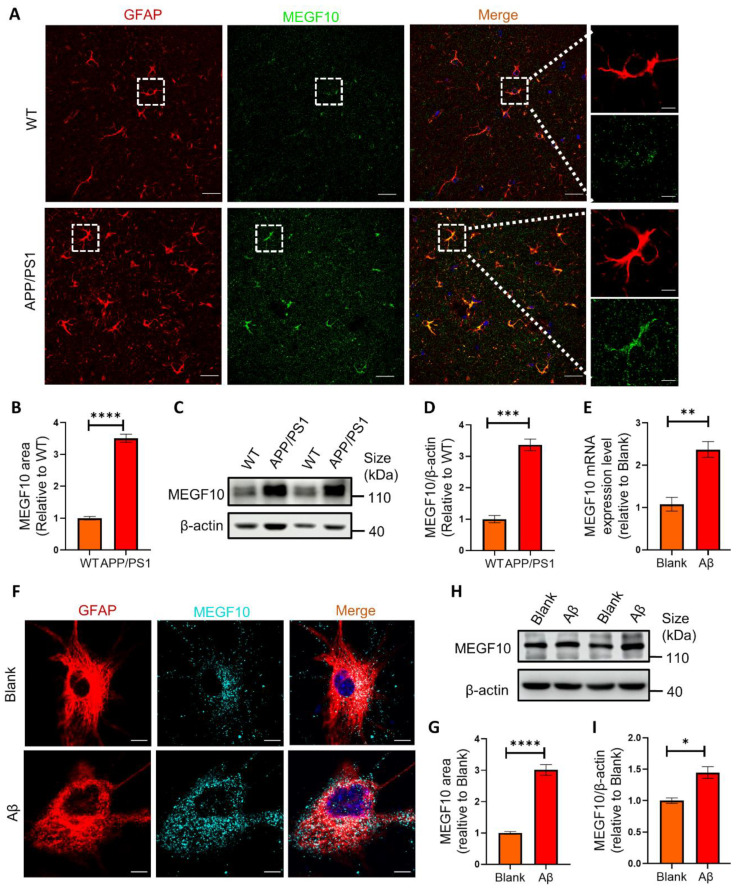
MEGF10 level is significantly increased both in vivo and in vitro. (**A**) Representative images of MEGF10 (green) in astrocytes (GFAP^+^, red) in APP/PS1 mice and WT mice. Scale bar: 25 μm. Representative astrocytes in white rectangles are magnified. Scale bar: 5 μm. (**B**) Quantification of MEGF10 area in APP/PS1 mice and WT mice by Image J. *n* = 5 mice per group. (**C**) Western blots analysis of MEGF10 in the hippocampal homogenates from APP/PS1 mice and WT mice. (**D**) Quantification of protein bands in (**C**) by ImageJ. Data are pooled from three independent experiments. (**E**) Quantification of mRNA encoding MEGF10 via qPCR analysis. Data are pooled from three independent experiments. (**F**) Representative images of MEGF10 (cyan) in the primary hippocampal astrocytes (GFAP^+^, red) after AβOs treatment. Scale bar: 5 μm. (**G**) Quantification of MEGF10 area in astrocytes in (**F**) by Image J. (**H**) Western blots analysis of MEGF10 in primary astrocytes challenged with AβOs. (**I**) Quantification of protein bands in (**H**) by ImageJ. Data are pooled from three independent experiments. Data in (**B**,**D**,**E**,**G**,**I**) are mean ± SEM, and an unpaired *t* test with two-tailed was used for statistical analysis. *, *p* < 0.05; **, *p* < 0.01; ***, *p* < 0.001; ****, *p* < 0.0001.

Previous studies have shown that phagocytic receptor MEGF10 in astrocytes is involved in synapse elimination during developmental and adult stage in the brain [6,12]. When MEGF10 was blocked, astrocytes displayed approximately 50% reduction in the synapse engulfment ability, suggesting that MEGF10 is one of the major phagocytic receptors for astrocytes to eliminate synapses [6,22]. But it’s remaining unclarified whether phagocytic receptor MEGF10 in astrocytes is involved in synapse elimination in APP/PS1 mice. In order to determine the association of synapse engulfment by astrocytes with MEGF10 receptor in APP/PS1 mice, we detected MEGF10 in GFAP^+^ astrocytes by IHC. Our results showed a significant increase in MEGF10 level in astrocytes of APP/PS1 mice (Figure 5A,B). Consistently, our Western blots results indicated that the protein level of MEGF10 was significantly increased in the hippocampus of APP/PS1 mice (Figure 5C,D). To further investigate the effect of Aβ on MEGF10 expression, we added AβOs to primary hippocampal astrocytes culture, and our quantitative real-time PCR (q-PCR) results showed that AβOs treatment significantly increased Megf10 mRNA transcript (Figure 5E). Consistently, our immunocytochemistry (ICC) assay results (Figure 5F,G) and Western blot results (Figure 5H,I) also showed that MEGF10 protein level was significantly upregulated in the cultured astrocytes with AβOs treatment. Taken together, these data demonstrated that MEGF10 level was remarkably increased in a microenvironment with Aβ in vitro and in vivo, which may contribute to the excessive phagocytosis of synapses.

### 2.6. The Colocalization of MEGF10 and Engulfed Synapses in Astrocytes

**Figure 6:**
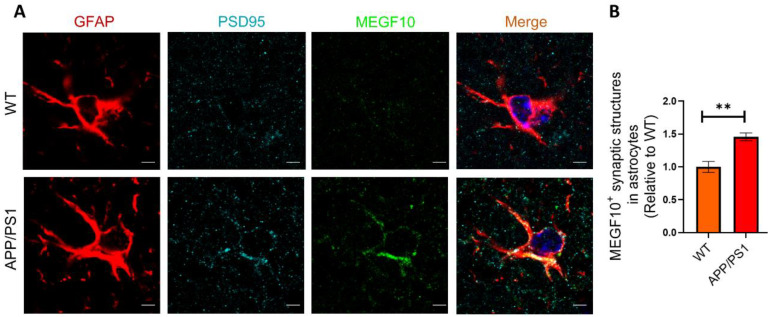
The internalized synapses were colocalized with MEGF10 in astrocytes. (**A**) Representative images of PSD95 puncta (cyan), MEGF10 (green) in astrocytes (GFAP^+^, red) in APP/PS1 mice and WT mice. Scale bar: 5 μm. (**B**) The level of engulfed PSD95 puncta colocalized with MEGF10 in astrocytes in (**A**). *n* = 5 mice per group. Data are mean ± SEM, and an unpaired *t* test with two-tailed was used for statistical analysis. **, *p* < 0.01.

To further investigate the relationship between MEGF10 and synapses, we detected MEGF10 and PSD95 in GFAP^+^ astrocytes in APP/PS1 mice. The results demonstrated that PSD95 was highly colocalized with MEGF10, further suggesting that MEGF10 excessively mediated synapse engulfment by astrocytes (Figure 6A,B).

## 3. Discussion

AD, the most common type of dementia, leads to a severe and progressive cognitive impairment, for which there is no effective treatment or cure. Synapse loss is an important contributor to cognitive decline in AD and begins long before the appearance of the amyloid plaques that characterize the initiation and development of AD. Patients with AD have a remarkable synapse loss, which is strongly associated with the loss of neurons, dendrites, dendritic arborization and the formation of tangles [20,23]. However, the activation of the complement cascade and the subsequent initiation of microglial synapse engulfment may contribute to the early stages of AD [4,24]. Moreover, synapse loss can precede tangle formation in animal models [25].

Astrocytes powerfully control the formation, maturation, function, and elimination of synapses through various secreted and contact-mediated signals [6,8,12,26,27,28]. Decades of pathological and physiological studies have focused on neuronal abnormalities in neurological disorders, but it is becoming increasingly evident that astrocytes are also important players in the disorders, such as amyotrophic lateral sclerosis, AD, Huntington’s disease and Parkinson’s disease, which are associated with synaptic defects [29,30,31]. Astrocytes can engulf cellular debris, such as synapses, axons, and apoptotic cells and can also engulf Aβ through phagocytic receptor MEGF10 [32,33,34]. Studies have demonstrated that astrocytes eliminate unnecessary excitatory synaptic connections in the adult hippocampus through MEGF10, and that this astrocytic function is crucial for maintaining circuit connectivity and thereby supporting cognitive function [12]. It is reported that astrocytes contact and eliminate synapses in a C1q-dependent manner, which compensates for microglial dysfunction and thereby contribute to pathological synapse loss in AD [35]. A recent report demonstrated that astrocytic synapse ingestion was increased by milk fat globule epidermal growth factor 8 (MFG-E8) in AD brain tissue relative to controls [36]. But it has also been shown that Aβ impairs the phagocytosis of dystrophic synapses by astrocytes in AD [37]. In this study, our findings demonstrated that astrocytes excessively engulfed synapses mediated by MEGF10 in APP/PS1 mice compared with wild-type controls. In AD, excessive phagocytosis of synapses by astrocytes could be beneficial for clearing dysfunctional or dead synapses, or it would be harmful for removal of functional synapses. But the beneficial or harmful contributions of astrocytes for synaptic phagocytosis varies at different stages of the disease and at different parts of the brain. We speculated that the amyloid beta pathology drove astrocytes to over-phagocytose synaptic terminals. An interesting question is that what is the relationship between the phagocytic Aβ and phagocytic synapse. In microglia, there is evidence that when microglia phagocytose Aβ bound on synapses, synapses are also phagocytosed [38], which demonstrates that microglial beneficial action through clearance of pathological protein may be linked to the potentially detrimental effect of synapse removal. Whether astrocytic clearance of Aβ is accompanied with increased removal of synapses remains to be explored.

Previous researches suggest that astrocytes participate in synaptic pruning through different pathways under different physiological and pathological conditions: MEGF10 and MERTK pathways in developing and adult brain [6], type 2 inositol 1,4,5-trisphosphate receptor (IP3R2) pathways in developing brain [39], ATP-binding cassette transporter A1 (ABCA1) pathways during the later stage of ischemia [33] and MFG-E8 receptors in AD brain. MEGF10 is an astrocyte-expressed receptor for phagocytosis that binds exposed phosphatidylserine (PS) via its ligands Gas6 and Protein S to mediate engulfment of synapses under physiological conditions [6,12]. MEGF10 has been shown to bind C1q which readily bind to synapses. Therefore, MEGF10 might also be involved in astrocytic synapse eating through complement pathway [40]. Our present results indicated that astrocytic MEGF10 level was significantly increased in APP/PS1 mice and cultured astrocytes challenged with AβOs, which was also consistent with the previous findings that Aβ_1-42_ elevated postsynaptic components inside astrocytes and decreased neuronal dendritic spine density [41]. Moreover, our results showed that MEGF10 was colocalized with the synapses in astrocytes, further suggesting that MEGF10 may contribute to the astrocytic synapse ingestion in APP/PS1 mice. A recent study demonstrated that MFG-E8 receptor played a role in synapse ingestion in AD brain, and blocking MFG-E8 reduced synapse phagocytosis by cultured astrocytes [36]. Further studies should be performed using MEGF10-knockout mice or human post-mortem samples or human iPSC derived organoid samples to investigate whether astrocytes participate in synapse elimination through MEGF10 in AD. Previous strategies to attenuate microglial phagocytic activity have shown some potential to reduce AD-associated pathology in mice, notably preventing synapse loss and rescuing cognitive deficits by complement C3 deficiency, C1qa knockout, or blocking [4,21,42].

**Figure 7:**
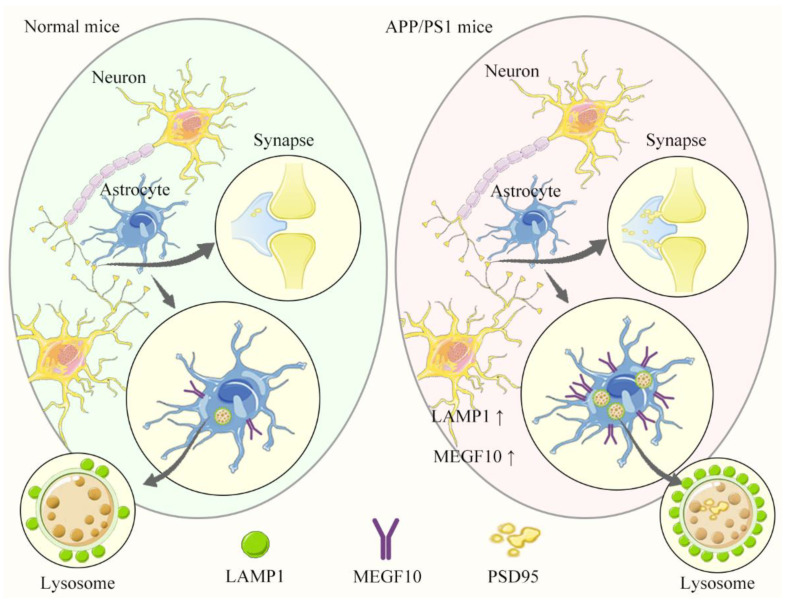
Schematic representation of astrocyte engulfing synapses excessively in a mouse model of Alzheimer’s disease. (**Left**) In the brain of normal mouse, astrocytes eliminate excessive synaptic connections to establish precise connectivity in neural networks. Lysosome-associated membrane protein 1 (LAMP1) and phagocytic receptors multiple EGF-like domains 10 (MEGF10) are expressed at low level in astrocytes of normal mouse. (**Right**) In the brain of APP/PS1 mouse, the levels of MEGF10 and LAMP1 are increased in astrocytes, resulting in excessive engulfment of synapses. Correspondingly, the astrocytic lysosomes contain PSD95 puncta is significantly increased.

In summary, we found here that astrocytes excessively engulfed synapses in APP/PS1 mice via increased MEGF10. The engulfed synapses were digested via lysosome pathway (Figure 7). The strategies targeting synapse engulfment pathway in astrocytes would be a novel approach to develop therapeutic agents for AD treatment.

## 4. Methods

### 4.1. Animals

Six-month-old male APP/PS1 transgenic mice and six-month-old male C57BL/6 mice were purchased from Beijing HFK Bioscience Co., Ltd. (Beijing, China). All mice were group-housed (3–5 mice per cage) with ad libitum access to food and water and a 12 h light/dark cycle (lights off at 7 p.m.) under constant conditions (22 ± 2 °C; 45% ± 10% humidity). All animal experiments were approved by the Institutional Animal Care and Use Committee of Tsinghua University, and adhered to the Guide for the Care and Use of Laboratory Animals of the Chinese Association for Laboratory Animal Science.

### 4.2. Antibodies

In this study, IHC, ICC and Western blots assays utilized the following primary antibodies: Mouse anti-β-actin antibody (Abcam, #ab8226, Cambridge, UK), Mouse anti-GFAP antibody (Cell Signaling Technology, #3670S, Boston, MA, USA), Goat anti-PSD95 antibody (Abcam, #ab12093), Rabbit anti-synaptophysin antibody (Abcam, #ab32127), Rabbit anti-LAMP1 antibody (Abcam, #ab24170), Rabbit anti-MEGF10 antibody (Sigma-Aldrich, #ABC10, St. Louis, MO, USA).

### 4.3. Preparation of AβOs

The Aβ_42_ Peptide (Chinese Peptide Company, #AMYD-003B, Hangzhou, China) was dissolved in 100% hexafluoroisopropanol (HFIP) to a final concentration of 1 mg/mL. After sonication in a water bath for 10 min, it was aliquoted into microcentrifuge tubes, and then the solvent was evaporated and the Aβ_42_ was stored at −20 °C. Immediately prior to use, the HFIP-treated Aβ_42_ was dissolved in dimethyl sulfoxide (DMSO) to 3 mg/mL, and diluted to 100 μM in PBS (20 mM phosphate buffer saline, pH7.4). Then the mixture was incubated at 37 °C for 4 h to form AβOs.

### 4.4. Primary Astrocytes Cultures

For primary astrocytes cultures, dissociated hippocampi cells from P0-1 mice were plated in T75 flasks and grown in DMEM (Dulbecco’s modified Eagle’s medium, Gibco, Thermo Fisher Scientific, Waltham, MA, USA) containing 5% fetal bovine serum (*v*/*v*), 5% horse serum (*v*/*v*), 100 U/mL penicillin, and 100 mg/mL streptomycin (Gibco) until they were confluent. The medium was changed every 2 days. To purify astrocytes from cultures, the cells were subjected to 12 h of continuous shaking to remove the microglia and oligodendrocytes at 7–10 days after plating. The supernatant containing the microglia and oligodendrocytes was discarded and the adhered astrocytes were subsequently washed. The cultures were treated with trypsin and the disassociated cells were re-plated in 12-well plates. The cultured astrocytes were regarded as mature after being cultured for 14 days. To investigate the effect of AβOs on the expression of MEGF10 receptor, the primary astrocytes were treated with 1 μM AβOs for 12 h, and then the cells were harvested for Western blots, ICC and q-PCR assay.

### 4.5. Immunocytochemistry (ICC)

Cells were washed with PBS three times, fixed in 4% paraformaldehyde (PFA) for 20 min at room temperature and permeabilized with 0.3% Triton X-100 for 30 min and blocked with 10% donkey serum albumin (DSA) in PBS for 1 h at room temperature. Then cells were incubated with primary antibodies overnight at 4 °C, followed by corresponding fluorescently-conjugated (−488, −594 or −647) secondary antibodies for 45 min and counterstained with Hoechst (1:10,000) for 15 min at room temperature in dark, and then mounted on coverslips with anti-fade mounting medium. Fluorescence signals were captured on a laser scanning confocal microscope (Leica TCS SP8, Wetzlar, Germany).

### 4.6. Immunohistochemistry (IHC)

Mice were anesthetized and perfused with ice-cold PBS containing heparin (10 U/mL) before sacrificed. Mouse hippocampus was immediately removed and divided along the sagittal plane. The left brain hemisphere was fixed in 4% PFA at 4 °C overnight and processed for paraffin-embedded sections. For immunohistochemistry analysis, 15 μm coronal paraffin-embedded serial sections were deparaffinized and subjected to antigen retrieval using citrate buffer (0.01 M, pH 6.0, 0.05% Tween-20) at 95 °C for 20 min. The sections were then incubated with 3% H_2_O_2_ and washed 3 times with 1× PBS. Sections were then permeabilized and blocked with 10% goat serum or donkey serum in 0.3% Triton-X 100 for 1 h at room temperature. Then sections were incubated with anti-PSD95, anti-GFAP, anti-MEGF10, anti-LAMP1 antibodies, followed by corresponding secondary antibodies conjugated to Alexa Fluor 488, -594 or -647, respectively. The sections were imaged on the Leica TCS SP8 confocal microscope. All images were analyzed by ImageJ Software (National Institutes of Health, Bethesda, MD, USA, Version 1.52a). For astrocytes engulfment assays, background subtraction was performed using ImageJ, then Imaris software (Bitplane, Belfast, Northern Ireland, UK, Version 9.8) was used to perform 3D reconstruction.

The following primary antibodies were used for IHC and ICC assay in this study: Mouse anti-GFAP antibody (Cell Signaling Technology, #3670S, 1:200), Goat anti-PSD95 antibody (Abcam, #ab12093, 1:200), Rabbit anti-synaptophysin antibody (Abcam, #ab32127, 1:500), Rabbit anti-LAMP1 antibody (Abcam, #ab24170, 1:200), Rabbit anti-MEGF10 antibody (Sigma-Aldrich, #ABC10, 1:200).

### 4.7. Brain Lysate Preparation

The mouse hippocampus tissues were homogenized in RIPA lysis buffer (MedChemExpress, #HY-K1001, Monmouth Junction, NJ, USA) containing phosphatase inhibitor cocktails (Solarbio Left Sciences, #P1260, Beijing, China) and protease inhibitor cocktail set I (Millipore, #539131, Burlington, MA, USA) using Tissue LyserII (QIAGEN, Hilden, Germany) and then centrifuged at 15,000× *g* for 30 min at 4 °C to collect the supernatant (RIPA-soluble fraction). The pellets were resuspended in guanidine buffer (5.0 M guanidine-HCl/50 mM Tris-HCl, pH 8.0) and centrifuged at 15,000× *g* for 1 h at 4 °C to obtain supernatants containing insoluble proteins (RIPA-insoluble fraction). The protein concentrations of soluble and insoluble fractions were determined using the BCA protein assay (Thermo Fisher Scientific, #23225, Waltham, MA, USA) according to the manufacturer’s instructions.

### 4.8. Western Blots

Protein samples from mouse hippocampus tissues or cell lysates were separated using 10–12% SDS-PAGE gels (Invitrogen, Frederick, MD, USA) and transferred onto nitrocellulose membranes (Merck Millipore, Billerica, MA, USA). After blocking with 5% non-fat milk for 1 h at room temperature, the membrane was incubated with the corresponding primary antibodies overnight at 4℃. Then HRP-conjugated secondary antibodies were applied at a concentration of 1:5000 for 1 h at room temperature. The bands in immunoblots were visualized by enhanced chemiluminescence using an Amersham imager 680 imaging system (GE Healthcare, Pittsburgh, PA, USA) and quantified by densitometry and Image J software.

The following primary antibodies were used for Western blots assay in this study: Mouse anti-β-actin antibody (Abcam, #ab8226, 1:1000), Rabbit anti-LAMP1 antibody (Abcam, #ab24170, 1:1000), Rabbit anti-MEGF10 antibody (Sigma-Aldrich, #ABC10, 1:1000).

### 4.9. RNA Extraction and Quantitative PCR (q-PCR)

Total RNA from cell lysates was extracted using TRIzol reagent. Reverse transcription was performed by EasyQuick RT MasterMix (Cwbio, #CW2019M, Taizhou, China) according to the manufacturer’s instructions. Relative gene expression of the cDNA was detected by real-time q-PCR using the 7500 Fast Real-Time PCR System (Applied Biosystems, Foster city, CA, USA) and SYBR Select Master Mix (Applied Biosystems, #4472908). Expression levels of the target genes were normalized to β-actin. Sequences used were: β-actin, 5′-TGTGATGGTGGGAATGGGTCAG-3′, and 5′-TTTGATGTCACGCACGATTTCC-3′, Megf10, 5′-TACCGCCATGGGGAGAAAAC-3′ and 5′-TTATCAGCGCAGTGAGGGAC-3′.

### 4.10. Statistical Analysis

Statistical testing was performed using Prism (GraphPad Software, La Jolla, CA, USA, Version 8.0). For comparisons between groups, whether the data were normally distributed was first determined using the Shapiro-Wilk test (Sigma-Plot). If data were normally distributed, an unpaired *t* test with two-tailed *p* values was used. If not, Mann-Whitney test was used. Results were expressed as group mean ± SEM, and *p* < 0.05 was considered statistically significant. *, *p* < 0.05; **, *p* < 0.01; ***, *p* < 0.001; ****, *p* < 0.0001; ns, not significant.

## Data Availability

The data in this study are available from the corresponding author upon reasonable request.

## Abbreviations

ABCA1	ATP-binding cassette transporter A1
AD	Alzheimer’s disease
Aβ	Amyloid beta
AβOs	Amyloid beta oligomers
CNS	Central nervous system
DMEM	Dulbecco’s modified Eagle’s medium
DMSO	Dimethyl sulfoxide
DSA	Donkey serum albumin
GFAP	Glial fibrillary acidic protein
HFIP	Hexafluoroisopropanol
ICC	Immunocytochemistry
IHC	Immunohistochemistry
LAMP1	Lysosomal-associated membrane protein 1
MEGF10	Multiple EGF-like domains 10
MERTK	Macrophage c-mer tyrosine kinase
MFG-E8	Milk fat globule epidermal growth factor 8
PBS	Phosphate buffer saline
PFA	Paraformaldehyde
PS	Phosphatidylserine
PSD95	Postsynaptic density-95
q-PCR	Quantitative real-time PCR
RNA	Ribonucleic acid
SDS	Sodium dodecyl sulfate
SEM	Standard Error Mean
SYN	Synaptophysin
WT	Wild-type
